# Computational discovery of natural medicines targeting adenosine receptors for metabolic diseases

**DOI:** 10.3389/fphar.2025.1671415

**Published:** 2025-09-02

**Authors:** Peng Wang, Zhiyu Xu, Yuqing Deng, Huiping Yuan, Yale Wu, Jing Jiang, Zhaohui Lyu, Zejun Li

**Affiliations:** ^1^ School of Electronic Information, Hunan First Normal University, Changsha, China; ^2^ College of Computer Science and Electronic Engineering, Hunan University, Changsha, China; ^3^ Wenzhou University of Technology, Wenzhou, China; ^4^ School of Computer Science, Hunan Institute of Technology, Hengyang, China

**Keywords:** metabolic diseases, adenosine receptors, natural medicines, computational strategy, artificial intelligence

## Abstract

Metabolic diseases—including type 2 diabetes, obesity, non-alcoholic fatty liver disease, and certain cancers—pose major global public health challenges. These conditions share common mechanisms such as insulin resistance, chronic inflammation, and oxidative stress. Although medical advances have improved disease management, current treatments remain suboptimal. Natural medicines have gained increasing interest due to their safety, bioactivity, and diverse mechanisms. This study targets adenosine receptors (ARs), key regulators in glucose metabolism, lipid homeostasis, and cellular stress. As members of the G protein-coupled receptor (GPCR) family, ARs include four subtypes—A1, A2A, A2B, and A3—each with distinct pharmacological profiles. We developed a multimodal computational strategy to design natural drug candidates that simultaneously target A1 and A2A, using A2A-selective ligands as controls to explore subtype selectivity. To mitigate toxicity, we incorporated a filtering criterion for low hERG channel affinity. A random forest-based QSAR model was constructed using SMILES representations to predict compound activity. A stacked LSTM neural network was applied to generate plant-derived molecules, while reinforcement learning and Pareto optimization enabled multi-objective refinement. Evolutionary operations—crossover, mutation, and selection—were further introduced to enhance molecular diversity and performance. The proposed framework successfully generated compounds with high target selectivity, low toxicity, and it has good drug-likeness and synthetic accessibility. This work presents a robust and intelligent strategy for natural drug discovery in metabolic diseases and underscores the promising synergy between botanical medicine and artificial intelligence in therapeutic innovation.

## 1 Introduction

Metabolic diseases, such as type 2 diabetes (DeFronzo et al., 2015; Ahmad et al., 2022), obesity, non-alcoholic fatty liver disease and certain types of cancer, have become major global public health challenges due to their high incidence and complex pathological mechanisms. These diseases are often accompanied by metabolic homeostasis disorders such as insulin resistance, chronic inflammation and oxidative stress. Long-term reliance on traditional drug treatments has limitations in terms of efficacy and safety. Natural medicines, with their diverse structures, strong biological activities and wide range of action mechanisms, have shown unique advantages in the intervention of metabolic diseases. However, the traditional process of natural drug discovery is time-consuming and has weak target specificity, which limits the efficiency of clinical transformation (Atanasov et al., 2021). With the development of artificial intelligence and computational pharmacology, molecular generation methods combined with multi-objective optimization strategies are becoming important means for efficient screening and design of natural drug candidates (Vamathevan et al., 2019). Against this background, targeting key receptors involved in glucose metabolism, lipid regulation and cellular stress, such as adenosine receptors, provides new targets and research paths for the precise design of natural drugs.

In the field of multi-target pharmacology, drugs can be combined with multiple specific targets at the same time, aiming to enhance the therapeutic effect and reduce the development of drug resistance (Wei et al., 2024a). Studies have shown that multi-target suppression of a partial target is often more effective than complete suppression of a single target, and this strategy is especially suitable for complex and multifactorial disease conditions (Fu et al., 2025; Zhou et al., 2024). Recent global–local re-interpretation of drug–protein interactions further corroborates this view, indicating that balanced modulation across multiple targets can enhance therapeutic efficacy in metabolic disorders (Zhou et al., 2024).In addition, when the multiple targets mentioned are actually multiple mutant forms of a single target, the drug also has the ability to bind to these mutants simultaneously (Hopkins, 2008).

In addition, since different proteins may share common structures with similar functions, this increases the risk of non-specific binding to non-target proteins (Cai et al., 2025; Wang et al., 2025). Therefore, when working to develop highly targeted drug molecules, it is important to enhance the targeting selectivity of drugs to ensure that they avoid improper binding to non-target proteins. The key to achieving this goal is to accurately identify and target the uniqueness of the target protein, thereby minimizing the non-targeted effects and improving the overall safety and effectiveness of the drug (Tan et al., 2025). In this context, deep learning and reinforcement learning technologies, with their ability to predict drug binding affinity to target proteins and evaluate potential non-targeted effects, show great potential and promise in facilitating highly targeted and selective drug design (Zeng et al., 2024). Developing highly targeted drugs has become a central goal in the field of drug discovery, which can not only greatly optimize the treatment of complex diseases, but also effectively avoid unnecessary side effects, resulting in safer and more efficient treatment options for patients. Recent heterogeneous-graph approaches, such as the interpretable multi-instance model for circRNA–drug sensitivity prediction (Niu et al., 2025) and the deep multi-instance framework for drug–disease associations (Gu et al., 2025), have further demonstrated the value of integrating multi-omics and network information in computational drug discovery”.

As early as the 1970s, statistical mathematical modeling based on perceptrons has been used for computer-assisted medicine. Design, this kind of mathematical method belongs to supervised machine learning. However, until now, unsupervised deep learning drug design research has begun to emerge. Among them, generative deep learning can break through the technical bottleneck of traditional computer-aided drug design by extracting hidden features from molecules. In Zhavoronkov et al. (2019) used a deep generation model to successfully discover high-quality lead compounds from target screening to nanomolar activity in just 46 days, marking a milestone in the application of generative deep learning to new molecular design (Xu et al., 2025).

The core of the program is focused on developing drugs that can bind efficiently to specific targets. adenosine receptors (AR)(Ijzerman et al., 2022), as a class of receptors similar to G protein-coupled receptors (GPCRS)(Weis and Kobilka, 2018), are endogenous to adenosine (Wang et al., 2023). Adenosine and AR are widely distributed in various tissues of the human body, and their interaction triggers a wide range of physiological and pathological processes. The AR family consists of four subtypes, A1, A2A, A2B and A3, each of which exhibits unique pharmacological properties, tissue distribution patterns and effect coupling mechanisms (Fredholm, 2010; Chen et al., 2024). This project will focus on drug molecules that generate Adenosine receptor A1 and Adenosine receptor A2A, as well as drug molecules that only affine A2A while serving as control affinity A1. In addition, to reduce the risk of toxicity and adverse events, drugs should be designed to exhibit low affinity for member H 2 of the potassium voltage-gated channel subfamily (often referred to simply as the hERG channel, or human Ether-a-go-go-Related Gene Channel) (Cai et al., 2021). This can effectively prevent the drug from binding with non-target.

## 2 Methods

### 2.1 Data sets and molecular selection

The ChEMBL database (Gaulton et al., 2011) is a publicly available drug molecule database developed by the European Bioinformatics Institute (EBI) in collaboration with pharmaceutical companies and other partners. It contains a large number of small molecule compounds and their biological activity data, including the biological activity and pharmacological properties of the compounds, the structure and chemical information of the compounds, the structure and physiological function of biomolecules and other data (Zdrazil et al., 2023). These data are derived primarily from journals and papers, partner data, and are integrated with data on today’s approved drugs, current clinical development candidates in medicine, and other public databases. Together, it brings together chemical and biological information from multiple sources, covering multiple species and multiple disease domains. If the information in the ChEMBL database is fully utilized, it can help deep learning models for drug screening, design, and optimization.

At present, the latest version of the ChEMBL database is the ChEMBL35 database updated in December 2024 (Zdrazil, 2025). In order to ensure the reliability of the data, we need to reasonably control the number of data sets to meet the specific needs of the research, we choose ChEMBL34 as the data set for this research. ChEMBL34 contains data on approximately 2.4 million unique drug-like compounds and over 20 million bioactivity data points related to these compounds. After downloading the database file, the data set needs to be preprocessed: For charged molecules, the charge should be standardized, metals, small molecules and super-large molecules removed, the entire data set should be checked, and duplicate data should be removed. Finally, a total of about 2.1 million data were obtained, which was used as the ChEMBL data set for pre-training of the generation model, so that the generation model could generate legitimate drug molecules. Data preprocessing can be implemented using RDKit.

The molecules selected in this study have corresponding CHEMBL ID in ChEMBL: adenosine receptor A1 is CHEMBL226, adenosine receptor A2A is CHEMBL251, and hERG channel is CHEMBL240 (Mendez et al., 2018). By extracting CHEMBL ID, about 23,000 ligands that are biologically active to the above molecules are extracted from the processed ChEMBL data set and constructed into LIGAND data set for fine-tuning the generation model.

In order to concretely quantify the biological activity of drug molecules, the ChEMBL database also provides PCHEMBL VALUE (in the ChEMBL database, the value is given by the negative logarithm of IC50, EC50, XC50, AC50, Ki, Kd, Potency). (hereinafter referred to as pX value) for reference (Lenselink et al., 2017). In relevant studies, the threshold of biological activity was defined as pX=6.5 . If the pX value of a molecule is lower than 6.5, it can be determined that the molecule lacks affinity for a specific target or has only a low affinity, that is, it does not meet our needs. Conversely, if the pX value is equal to or higher than 6.5, it indicates that the molecule has a high affinity.

### 2.2 Deep learning

Today’s deep learning models show excellent performance in many areas, including widely used predictive models and generative model structures (Wei et al., 2024b). Predictive model and generative model are two important application directions of deep learning in drug molecule discovery. The predictive model is mainly trained for predictive analysis of a given molecule, including predicting the biological activity, drug efficacy, toxicity and other information of the molecule (Lai et al., 2025). Generative models automatically synthesize new molecular structures through deep learning models, providing important support for the design of new drugs (Wang et al., 2024).

**FIGURE 1:**
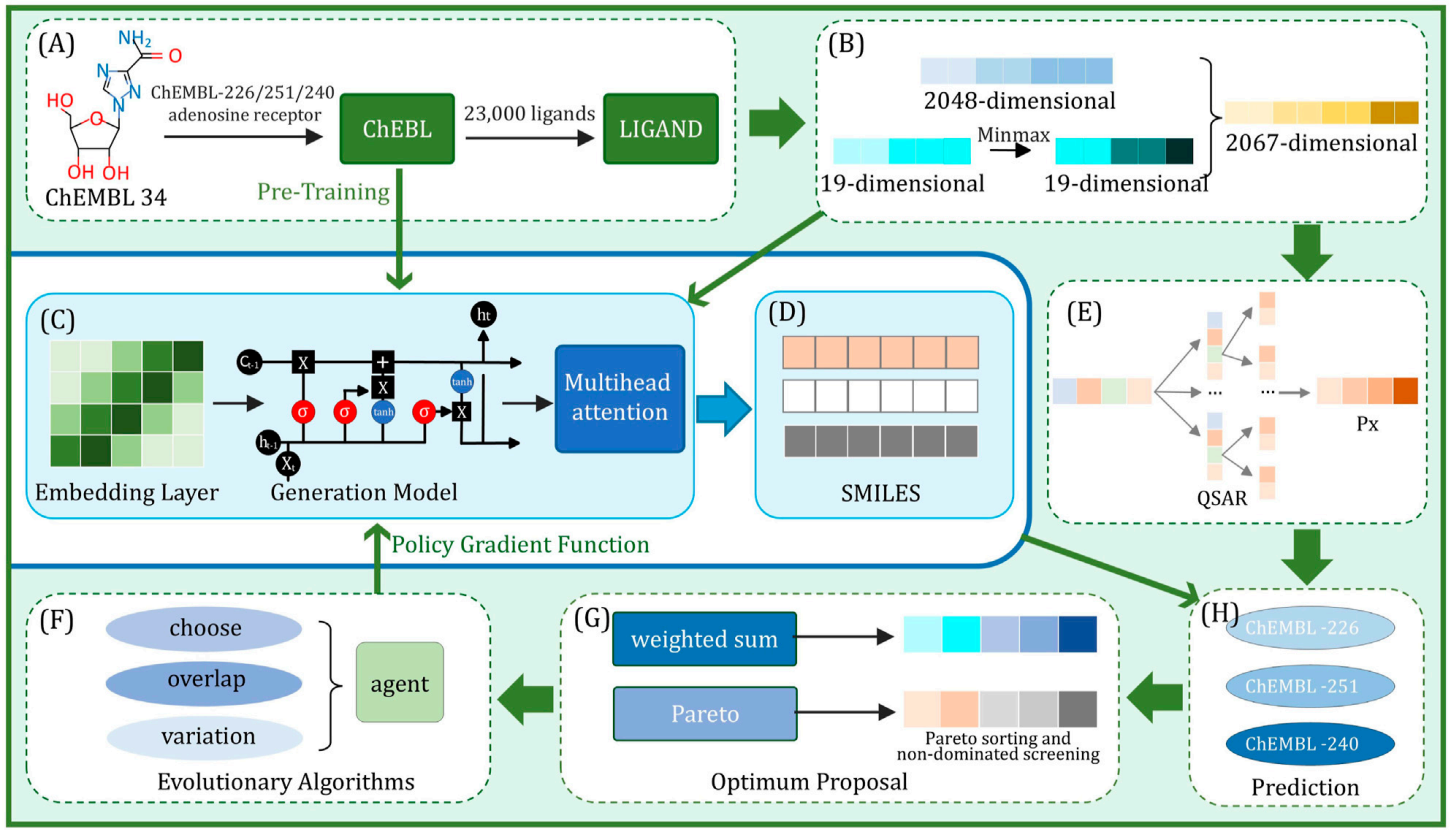
The computational flowchart of natural drugs for adenosine receptors. Arrows indicate process flow, and elements are labeled from **(A-H)**.

In this project, the predictive model is trained first, aiming to make it have the ability to calculate pX value for a given molecule. By accumulating large amounts of training data and continuously optimizing the model, we can gradually improve the performance of the predictive model, and the biological activity of the molecule can be predicted more accurately. The generated model is then pre-trained and optimized using the strategy gradient and loss function to reduce the error rate of molecular generation and ensure that the generated molecule has the correct SMILES format. After pre-training, the generative model already has some generative ability, but it needs to be fine-tuned to the specific research task to ensure that the resulting molecules can meet the requirements of the research purpose. After fine-tuning the three target ligands of this research, the generative model can generate high-quality molecules suitable for research purposes. Through the above training and optimization, an efficient molecular generation and prediction model is successfully constructed, which is ready for the reinforcement learning. The process of reinforcement learning is shown in Figure 1, this diagram illustrates a chemical molecule data processing workflow, where the molecular structure is first converted into feature vectors, then normalized through Minmax scaling, fed into a QSAR model for prediction, and finally generates SMILES strings through multi-head attention and policy gradient functions.

#### 2.2.1 Prediction model

Quantitative Structure-Activity Relationship (QSAR) model is one of the commonly used prediction models in drug molecular development (Kiralj and Ferreira, 2009). The regression QSAR model uses a series of molecular descriptors to describe the physical and chemical structural properties of drug molecules with the help of mathematical means. By constructing linear or nonlinear correlations between the structure and activity of molecular compounds, the model can predict the pharmacodynamic activity of new drug molecules. Regression QSAR model has been widely used in drug discovery field. It can screen drug molecules in the early drug development stage, improve screening efficiency and reduce costs. In addition, by analyzing the construction relationship of the regression QSAR model, researchers can better understand the relationship between drug molecular structure and activity, and provide guidance for further drug design.

In this project, we adopted a regression QSAR model as a predictive tool to predict the pX value of each molecule generated for a specific target. To enhance the fault tolerance of the QSAR model and enable it to make predictions about more chemical molecules, we added low-quality data to the dataset with no pChEMBL value, i.e., molecules labeled as not biologically active or with no defined pX value. For these data, define the pX value of these data to be 3.99, which is slightly less than 4, thus eliminating the imbalance of the data set and ensuring that the trained regression QSAR model has the ability to predict negative samples. In the model-training phase, we explicitly addressed the class imbalance between positive ( pX>6.5 or pX=6.5 ) and negative ( pX<6.5 or undefined) samples by assigning sample weights: positives received a weight of 1.0, while negatives were down-weighted to 0.1 in both the Random-Forest loss and the reinforcement-learning reward, thereby preventing the model from being dominated by the larger negative set. In this way, the chemical diversity of acceptable molecules can be ensured without reducing the quality of the model.

Labels such as CHEMBL ID, SMILES, pX value, comment, Standard Type, Standard Relation are extracted from the ligand data set for target molecules, and pX value is set to 3.99 for low-quality data. For each molecule its ECFP6 fingerprint is calculated by the RDKit Morgan fingerprint algorithm (Rogers and Hahn, 2010), as an input to the predictive model, with 2048 bits (i.e., a vector with 2048 dimensions). In addition, in order to describe the properties of molecules, it is necessary to add a 19-dimensional physicochemical descriptor: Molecular weight, logP, number of hydrogen bond acceptors and donors, number of rotatable bonds, number of amide bonds, number of bridgehead atoms, number of heteroatoms, number of helix atoms, number of heavy atoms, SP3 The fraction of hybrid carbon atoms, the number of alicycles, the number of saturated rings, the number of total rings, the number of aromatic rings, the number of heterocycles, the number of valence electrons, the polar surface area, and the Wildman-Crippen MR Value.

Therefore, each molecule in the data set will be converted into a 2067-dimensional feature vector. Before being submitted to the prediction model, the values of these eigenvectors are normalized to the interval [0,1] using the MinMax method. The output of the prediction model is a probability value that evaluates the probability of a given compound based on whether the vector is valid.

The mainstream directions of QSAR (Trinh et al., 2022) models mainly include: Statistics-based QSAR model: This type of model is based on statistical analysis of molecular structure and activity data, and analyzes and predicts by establishing mathematical models. The advantage is that it is easy to understand and implement, but the disadvantage is that it cannot deal with more complex molecular structures and characteristics. Typical examples of these models include partial least squares regression (PLS) (Xie et al., 2022) and multiple linear regression (MLR) (Shams et al., 2021). QSAR model based on machine learning: This type of model uses machine learning algorithms to map molecular structure and activity data to a high-dimensional space, and describes the relationship between them by building a nonlinear model. Its advantage is that it can handle relatively complex molecular structures and features, but it requires a large number of data sets and computational resources. Typical examples of such models are Support Vector Machine SVM) (El Morr et al., 2022), Random Forest (RF) (Belgiu and Draguţ, 2016) QSAR model based on deep learning: This type of model utilizes deep neural network algorithms to construct efficient nonlinear mapping relationships through multi-level feature extraction and abstraction. It has the advantage of being able to process large-scale molecular structure and activity data, but requires more computational resources and expertise. At present, common Deep Neural networks (DNN) (Pan et al., 2012) and Multi-task Deep Neural networks (MT-DNN) developed on this basis belong to this category. According to the relevant research on the target molecules in this subject (Liu et al., 2021), and considering the training time of the model and the available computing power, we choose to use the random forest algorithm to build the regression QSAR model, set the number of trees to 1000, take the Gini index as the segmentation standard, and realize it through Scikit-Learn.

The prediction model, which extracts the required labels from the ligand dataset for the target molecule. Each molecule is transformed into a 2067-dimensional vector. Subsequently, a MinMax operation is performed to normalize the vector values to the range of [0,1]. This normalized vector is then input into the QSAR model based on random forests, ultimately yielding the probability of the compound’s activity based on this vector.

Random Forest’s built-in out-of-bag error provides an unbiased internal validation, which is particularly advantageous for our highly imbalanced positive/negative sample ratio. These empirical and practical considerations collectively led us to adopt Random Forest as the QSAR engine. Random Forest was selected over XGBoost because, on our imbalanced dataset, it yielded 4% lower MAE and 6% higher AUROC in 10-fold cross-validation while requiring 30% less tuning time.

#### 2.2.2 Generative model

Molecules characterized by the Simplified molecular Input Line input system (SMILES) (Weininger, 1988) are essentially sequences arranged according to specific rules, the hidden states that deep learning models want to learn are actually relationships between sequences, just like the objects that NLP problems deal with. Therefore, in order to learn the relationship of atoms within the entire drug molecule, which can be regarded as natural language text, using RNNs as a model for deep learning is a more appropriate choice. RNNs can accept SMILES strings as input, identify and understand the molecules represented by SMILES strings by examining them one by one, such as identifying the bonds, functional groups, etc., predict the next character, and continue the process to predict the entire molecule. Generative models build molecules in SMILES form, but generative models cannot generate molecules out of thin air and require us to provide SMILES dictionaries. Each drug molecule represented by SMILES in the CHEMBL and LIGAND data sets (Lagarde et al., 2015) is split into a series of markers, including bonds and roots. In this way, after processing all the data, we can extract all the markers that have appeared in the data set and collect all the markers that exist in the data set, thus forming the SMILES vocabulary of this topic. The final vocabulary contains 88 tokens, which are placed in order and the generative model is trained to form a valid SMILES sequence with the correct syntax.

The RNN model for SMILES sequence generation consists of six layers: an input layer, an embedding layer, three cyclic layers, and an output layer. After the drug molecule is represented as a sequence of characters, the RNN can receive it as a classification feature through the input layer. In the embedding layer, the vocabulary size is set to 88, consistent with the size of the SMILES vocabulary collected; The embedding dimension is set to 128, so that each drug molecule can be converted to a 128-dimensional vector. In the RNN model, Long Short-Term Memory (LSTM) (Van Houdt et al., 2020) is a commonly used cyclic unit. Compared with the traditional RNN model, LSTM can more effectively avoid the situation of gradient disappearance or local gradient explosion, thus improving the accuracy and generalization ability of the model. In addition, LSTM can better control the flow of information through Gated mechanisms and memory units, thereby improving the performance of the model, and compared to gated Recurrent units (GRUs) (Dey and Salem, 2017), it can effectively handle long-term dependencies and information transfer in string sequence data. Therefore, for the loop layer, we opt to use LSTM as the recurrent units, employing a 3-layer LSTM as the basic building block and stacking it up to 9 layers within the module of the generative model, while setting the number of hidden neurons to 512, instead of using GRU.In the output layer, the output for each position determines which character from the vocabulary is selected to increase the probability of SMILES strings.

Compared with ordinary language sentences, the length of SMILES molecules is obviously much longer than the character length of ordinary sentences. Therefore, in this topic, a single RNN model may not be able to learn the relationship between atoms in the whole molecule, and the training effect of using a single RNN model is relatively limited. If you want to improve training, the most straightforward way is to stack multiple RNNS, using the output and hidden state of the previous RNN model as the input of the next RNN model. In the forward propagator, the input is first renormalized to fit the dimensions of the embedding layer. Then, between each two layers of RNN model, the output of the RNN model is activated by Pytorch’s ReLU function, and the shape of the output is modified by a fully connected layer to the dimension size of the embedding layer, so that it can be used as the input of the next RNN model. In the training phase, we add a start flag (GO) to the front end of each data batch as an input signal, and set an end flag at the end of the same data batch. This ensures that the generation network can accurately select the appropriate label at each iteration based on the previously generated sequence information. For the loss function of RNN, we use the negative logarithmic likelihood function to build it to ensure that each marker in the output sequence is selected with maximum probability after training; At the same time, in order to optimize the model parameters, we used Adam algorithm instead of the traditional gradient descent process to optimize the loss function. In the training process of this subject, the learning rate is set to 10–3, the batch size is set to 512, and the training cycle is set to 1000.

#### 2.2.3 Self-attention mechanism

Self-attention mechanism (Yang et al., 2016) is a widely used technique in deep learning, which has some flexibility and can be customized for different tasks and data to better meet the needs of different scenarios. In the field of NLP problems, self-attention mechanisms are also widely used. In language modeling, translation, summary generation, sentiment analysis and other tasks, the self-attention mechanism can help the model better understand the relationship between different parts of the input data, thereby improving the performance and accuracy of the model.

Similarly, as mentioned above, due to the similarity between drug molecule discovery research and NLP problem, self-attention mechanism can also be applied in deep learning of this subject, and has objective performance improvement.

Self-attention mechanisms can help generative models better understand the relationships between different atoms in a molecule and deal with long-distance dependencies in molecules. In drug molecular design, drug molecules are usually composed of many atoms, and the interactions between these atoms are very complex, and the interactions between different atoms may be affected by other atoms in the molecule, and may even involve distant parts of the molecule, which is difficult to capture with traditional neural network models. In the previous article, we represented each molecule as a vector, and the self-attention mechanism can weight them according to the relationships between different atoms, so as to better capture and predict the interactions between different atoms in the molecule, understand the relationships between different parts of the molecule, and thus better design drug molecules with specific functions and properties.

In this project, the self-attention mechanism can be implemented through Pytorch’s multi-head attention module (nn.MultiheadAtention). According to the article named of Attention is all you need published by Vaswani et al. (2017), the calculation formula is defined as the following Formulas 1, 2:

$$\;MultiHead\left(Q,K,V\right)=Concat\left(head_{1},\cdot \cdot \cdot ,head_{h}\right)W^{O}$$ (1)

among that

$$\;headi=Attention\left(QW_{i}^{Q},KW_{i}^{K},VW_{i}^{V}\right)$$ (2)

And Attention is defined as the following Formula 3:

$$\;Attention\left(Q,K,V\right)=softmax\left(\frac{QK^{T}}{\sqrt{d_{k}}}\right)$$ (3)

In the multi-head attention module, the input of the module includes three tensors: query (Q), key (K), and value (V). These tensors are usually of the shape (seq-len, batch size, embed-dim), where seq-len denotes the sequence length, batch size denotes the batch size, and embed dim denotes the embedding dimension. Where $d_{k}$ is the dimension of the key vectors (here $d_{k}$ = 128).When the input query, key, and value are all the same vector or matrix, the multi-head attention module implements the self-attention mechanism. The multi-head attention module is applied in the forward propagation function of the generative model. Considering the limitation of training time and computing power, the self-attention mechanism is only applied in the output of the last layer of the stacked RNN model, rather than between each layer of RNN model. At the same time, it is necessary to consider whether the shape of the output layer is consistent with the input requirements of the multi-head attention module, otherwise it may have the opposite effect and lead to training failure. The prediction model and generation model are collectively called SNNMR model. The generative model process illustrates the architecture of a neural network model designed to generate SMILES strings. Initially, the input data is encoded through an Embedding Layer, followed by processing through a Long Short-Term Memory (LSTM) network to handle sequential data. Subsequently, the model employs Multi-Head Attention to capture relationships between different parts of the sequence, which includes Scaled Dot-Product Attention and Concatenation operations. Finally, after linear transformations, the model outputs the SMILES string, a text format used for representing molecular structures.

### 2.3 Strategy optimization

After the pre-training and fine-tuning of the prediction model and the generative model respectively, in order to strengthen the generation strategy of the generative model, we use the multi-objective optimization strategy (MOO) for reinforcement learning, so that it can maximize each objective in each scene.

Building SMILES molecules within the framework of reinforcement learning can actually be seen as a series of decision steps. The generation model generates a batch of SMILES by progressively sampling tokens based on calculated probabilities; The generation model generates a batch of SMILES strings by sampling them step by step according to the calculated probabilities. These valid SMILES strings are then parsed into molecular structures by the predictive model and further encoded into descriptors. From these descriptors, the model is able to calculate the predicted pX value; The predicted pX value is converted into a single value according to the multi-objective optimization strategy, which is used as a reward for each molecule; These SMILES molecular sequences and their rewards are sent back to a generative model for training using a strategy gradient approach. These four steps form the training cycle of reinforcement learning. In the reinforcement learning stage, the generative model and the predictive model can be viewed as decision and reward functions respectively.

#### 2.3.1 Object definition

In this topic, the objectives of reinforcement learning are defined as the following Formula 4:

$$\;maxmizeR_{1},maxmizeR_{2},\cdot \cdot \cdot ,maxmizeR_{n}$$ (4)

Among them, the target molecules of reinforcement learning in this subject are A1, A2A, hERG, and 3 molecules, that is, n is 3 in this subject. $R_{i}$ represents the score for each object i , calculated by the following Formula 5:

$$R_{i}=\left\{\begin{array}{cc} minmax\left(pX_{i}\right),\; & \text{if high a f finity required}\\ 1-minmax\left(pX_{i}\right),\; & \text{if low a f finity required}\\ 0,\; & \text{if SMILES invalid} \end{array}\right.$$ (5)

Where $pX_{i}$ represents the predicted score for the *i*th target molecule, which is normalized to the interval [0.1] as a reward score. If the target does not require agreeableness or low agreeableness, the score is subtracted from 1, the inverse. In this case, for both A1 and A2A, $R_{i}$ can be expressed the following Formulas 6, 7:

$$\left\{\begin{array}{c} R_{A1}=minmax\left(pX_{A1}\right)\;\\ R_{\mathrm{A2A}}=minmax\left(pX_{\mathrm{A2A}}\right)\;\\ R_{h_{\mathrm{ERG}}}=1-minmax\left(pX_{h_{\mathrm{ERG}}}\right)\; \end{array}\right.$$ (6)

In the case of no affinity to A1 and only affinity to A2A, $R_{i}$ can be expressed as:

$$\left\{\begin{array}{c} R_{A1}=1-minmax\left(pX_{A1}\right)\;\\ R_{\mathrm{A2A}}=minmax\left(pX_{\mathrm{A2A}}\right)\;\\ R_{h_{\mathrm{ERG}}}=1-minmax\left(pX_{h_{\mathrm{ERG}}}\right)\; \end{array}\right.$$ (7)

Minmax normalizes predicted $pX_{i}$ to [0,1] across the batch. To evaluate the performance of the generative model, we use three metrics to calculate the properties of the generative molecules: va-lidity, desirability, and uniqueness. It is calculated by the ratio of effective, desirable and unique molecules generated by the generative model to the total number of molecules in a training cycle. Among them, a valid molecule is defined as: if the molecule has a valid SMILES sequence, the generation of the molecule is considered to be effective; Desirable molecule is defined as follows: if the pX value of the molecule is greater than or equal to 6.5 (in actual implementation, all reward scores are greater than the threshold value, when pX=6.5 , reward score is 0.5, that is, the threshold value is 0.5), it can be identified as having biological activity for the target molecule, then the generation of the molecule is desirable; A unique molecule is defined as a molecule that is unique if it is different from other molecules in the dataset.

#### 2.3.2 Multi-objective optimization

For these three indicators, I use two MOO schemes to make decisions, namely, weighted scheme and Pareto optimization scheme. In the weight-based scheme, for the *i*th target molecule (total n = 3 in this subject), the weight wi of the *i*th target is determined according to the ratio of the number of generated molecules whose reward score is less than and greater than the threshold value, and its weight is defined the following Formula 8:

$$w_{i}=\frac{r_{i}}{\sum_{k=1}^{N}r_{k}}$$ (8)

Where N represents the total number of molecules generated, and $r_{i}$ is defined as the following Formula 9:

$$r_{i}=\frac{N_{i}^{l}}{N_{i}^{h}}$$ (9)

Where $N_{i}^{l}$ and $N_{i}^{h}$ represent the number of molecules whose scores are below and above the threshold, respectively, in the generated molecules. The final reward $R^{*}$ is defined as the following Formula 10:

$$R^{*}=\overset{n}{\underset{i=1}{\sum }}w_{i}R_{i}$$ (10)

In this way, in a training cycle, the generative model can set weights for target molecules according to this batch of generated molecules, so as to balance their contributions in the reward score, so as to achieve the purpose of multi-objective optimization.

In an optimization scheme based on Pareto frontier, given two solutions $m_{1}$ and $m_{2}$ whose scores are $\left(x_{1},x_{2},\ldots ,x_{n}\right)$ and $\left(y_{1},y_{2},\ldots ,y_{n}\right)$ , then only as the following Formula 11

$$\forall j\in \left\{1,\ldots ,n\right\}:x_{j}\geq y_{j}\text{ and }\exists j\in \left\{1,\ldots ,n\right\}:x_{j}>y_{j}$$ (11)

When $m_{1}$ dominates $m_{2}$ under the Pareto criterion, that is, $m_{1}$ dominates $m_{2}$ . Where $x_{j}$ is defined as the following Formula 12:

$$x_{j}=\left\{\begin{array}{cc} 1,\; & \text{if }R_{j}>t_{j}\\ \frac{R_{j}}{t_{j}},\; & \text{if }R_{j}\leq t_{j} \end{array}\right.$$ (12)

Among them, $t_{j}$ represents the threshold value of the JTH target molecule. As mentioned above, in this project, the threshold value of the three target molecules is set to 0.5. If the above conditions are not met, there is no dominant relationship between $m_{1}$ and $m_{2}$ .

After determining the dominant relationship among all solutions, a non-dominant sorting algorithm is used to obtain Pareto frontiers of different levels consisting of a set of solutions (Deb et al., 2000). The solution at the top is constrained by the other solutions at the bottom. After the frontier order from the dominant solution to another dominant solution is determined, we no longer limit ourselves to comparing the crowding distance between molecules within the same boundary, but order the molecules according to the average value of the local distance. Specifically, molecules with larger valley local distances will be assigned higher rankings. The final reward $R^{*}$ is defined as the following Formula 13:

$$R^{*}=\left\{\begin{array}{cc} 0.5+\frac{k-N_{\text{undesired}}}{2N_{\text{desired}}},\; & \text{if desired}\\ \frac{k}{2N_{\text{undesired}}},\; & \text{if undesired} \end{array}\right.$$ (13)

Where k represents the index of the solution in the Pareto sort. The final reward score for the desirable and undesirable solution is placed in the interval of (0,0.5) and (0.5,1) respectively, so that it can be separated.

For each step in the generation process, the generation model calculates the probability that each tag in the vocabulary is selected based on the sequence generated in the previous step. By applying the expected final reward obtained from the prediction model, its parameters are updated via the policy gradient. The objective function is as the following Formula 14:

$$J\left(\theta \right)=\overset{t=1}{\underset{T}{\sum }}\log G\left(y_{t}|y_{1:t-1}\right)\cdot R^{*}\left(t_{1:T}\right)$$ (14)

Maximizing this function optimizes the parameters in the generative model to ensure that, after the generative model is trained, it is able to construct the desired SMILES sequences that result in the highest reward score.

#### 2.3.3 Crossover, mutation and selection

In the above method, this paper constructed an executable neural network model for drug molecule design research. However, in the initial attempt, we found a problem: although the training effect was quite good, the molecules generated by the generative model began to show a trend of convergence after a relatively short training cycle: Within a training cycle, the molecules produced by the generative model are almost identical, with differences in only a few atoms, which is obviously unsatisfactory. To increase the diversity of generated molecules, we adopted the following strategies.

Evolutionary algorithms (EAs) (Vikhar, 2016) are a class of optimization techniques inspired by the mechanisms of biological evolution. By simulating natural selection processes, EAs iteratively select individuals with the highest fitness within a population and employ genetic operations such as crossover and mutation to generate new individuals, thereby continuously optimizing the objective function. In the field of deep learning, evolutionary algorithms have found widespread application, with genetic algorithms (GAs) and evolutionary strategy (ES) algorithms being particularly prominent. These methods are commonly used to optimize neural network architectures, structural weights, and hyperparameters, thereby enhancing model performance. Drawing inspiration from the work of Professor Xuhan Liu (Liu et al., 2021), this study leverages evolutionary algorithms to improve the diversity of generated molecules. Specifically, we adapt the core principles of selection, crossover, and mutation from EAs, with a modification that applies the selection step after crossover and mutation, prior to integrating these operations into the training of generative models. During the training process, three models—agent, prior, and crover—are employed. These models share the same Recurrent Neural Network (RNN) architecture and are initialized using pre-trained weights from the generative model, as well as fine-tuned weights saved during previous training iterations. The agent and crover models are loaded with the fine-tuned weights specific to the target molecule, where the agent’s parameters are derived from the most recent reinforcement learning checkpoint. In contrast, the prior model is initialized with pre-trained weights without fine-tuning. Throughout the reinforcement learning phase, the prior model remains static, with its parameters fixed, and serves solely as a variation factor to introduce diversity into the training process. During each training cycle, the agent model is updated based on reinforcement learning objectives, and its parameters are saved at checkpoints corresponding to the highest reward scores. At predefined intervals, both the agent and crover models synchronize their parameters with those of the saved optimal model to further refine training outcomes. This iterative process ensures continuous improvement in the quality and diversity of generated molecules, aligning with the overarching goal of optimizing molecular design through evolutionary-inspired deep learning techniques.

These three models come into play when reinforcement learning generates SMILES sequences: Set the cross-change rate to a random number θ(0,1) , and the threshold of cross-change is 0.5. When each character is generated, if θ>0.5 , the model hidden state is updated by the crossover model, that is, the generation of the character is determined by the crossover model, otherwise, it is determined by agent. In addition, a random number (0,1) is set as the mutation rate ϵ and the mutation threshold of 0.1. When each character is generated, if ϵ>0.01 , the character generation is determined by agent and crover; otherwise, it is determined by prior. Next, calculate the probability of each molecule being selected based on its score use the roulette wheel selection method to select the top 20% of molecules from all generated molecules according to these probabilities. The selected molecules are subsequently used to train the proxy network.

First, the selection operation is carried out. From the current population (i.e., the set of generated molecules), molecules on the Pareto front are preferentially screened based on optimization objectives. These molecules represent the top-ranked, high-quality solutions in the current multi-objective optimization problem.

Next, the crossover operation is implemented. The crover model (another model with molecule generation capabilities) is employed to mix the SMILES sequences of two parent molecules. Through this crossover and mixing process, it is expected to integrate the advantageous characteristics of the parent molecules and generate new molecules with novel properties.

Subsequently, the mutation operation is conducted. For the molecules selected after the crossover operation, local modifications are carried out. Specifically, operations such as atom substitution and fragment insertion can be employed to fine-tune the molecular structures, thereby further expanding the search space of molecules and increasing the likelihood of obtaining superior molecules. Finally, the new population generation operation is executed. The molecules obtained after a series of evolutionary operations, including selection, crossover, and mutation, are added to the training pool for the next round, providing a basis for subsequent iterative optimization. Throughout the evolutionary process, the crossover threshold is set to 0.5 to control the probability of crossover operations. Meanwhile, a random number within the range of (0, 1) is designated as the mutation rate ϵ , and the mutation threshold is set to 0.1. These parameter settings precisely regulate the degree and frequency of mutations during the evolutionary process. SMILES sequences generated in this way are fed into the strategy gradient function, computed by the predictive model and returned to the generated model to affect the parameter update of the model. In this way, we increase the uniqueness of the resulting molecule.

## 3 Experiments

**FIGURE 2:**
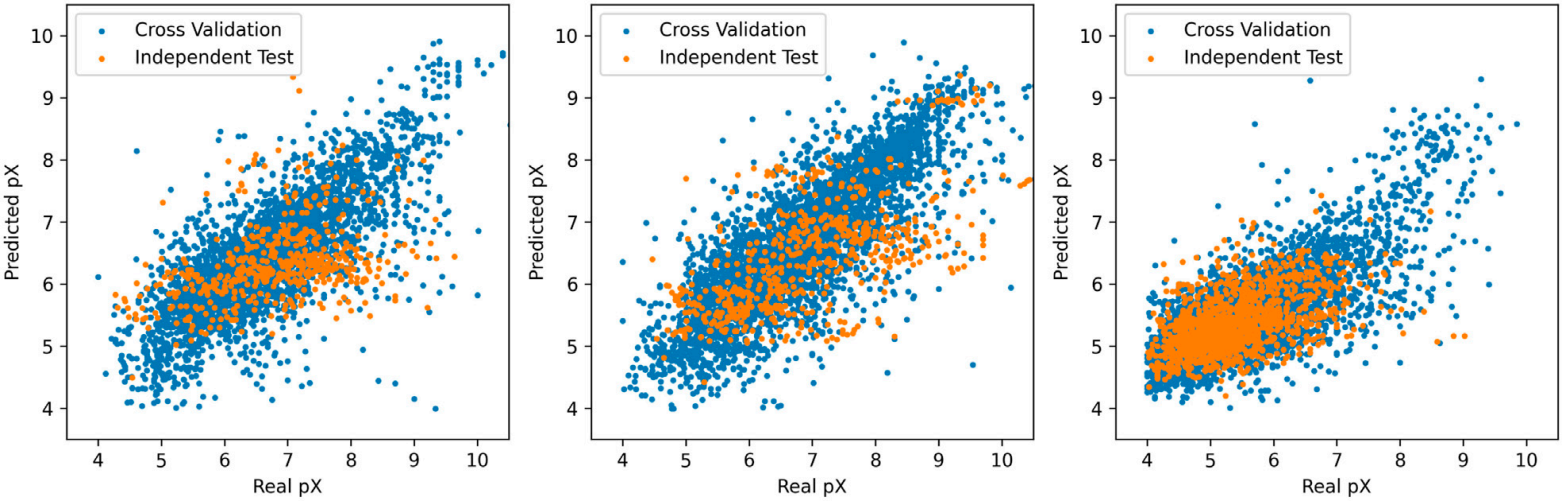
Prediction model scatter.

The training of the prediction model based on the random forest algorithm took about 15 h. After 1000 training cycles, the error rate of the prediction model has been reduced to less than 5 % , and it can be basically concluded that the pX value given by the prediction model is correct. All experiments were conducted on a cloud instance equipped with an 8 vCPU platform and an NVIDIA A10 GPU (24 GB VRAM). Training utilized CUDA 11.6 and PyTorch 1.13. The RF-QSAR model consumed about 15 GPU-minutes; the full reinforcement-learning pipeline (1,000 epochs) required about 36 h on the A10 GPU under mixed-precision (FP16) mode. In order to verify the accuracy of the prediction model, we also simply trained the support vector machine, multi-task deep neural network, K-nearest neighbor, least square regression and other models, and cross-verified the prediction model. As shown in Figure 2.

The three graphs from left to right show the relationship between the predicted pX values given by the prediction mmodel and the actual pX values for A1, A2A, and hERG ligands. Each point in the diagram represents a molecule for the target ligand, where the x coordinate represents the actual pX value and the left side of y represents the predicted pX value given by the prediction model. As can be seen from the figure, the random forest algorithm is generally consistent with other algorithms. Since the generative model may create a large number of novel molecules during the training process, these molecules are significantly different from the samples in the training set. In order to ensure the robustness and fault tolerance of the prediction model, random forest algorithm becomes a better choice because of its advantages.

### 3.1 The feasibility and effectiveness

In the training process of generating the model, we mainly judge the quality of the model training effect through the feasibility and effectiveness, and independence is mainly used in the internal training of the model and the gradient strategy. Whether the molecules generated by the generative model are effective, whether they are biologically active and have the affinity we need, that’s what matters.

**FIGURE 3:**
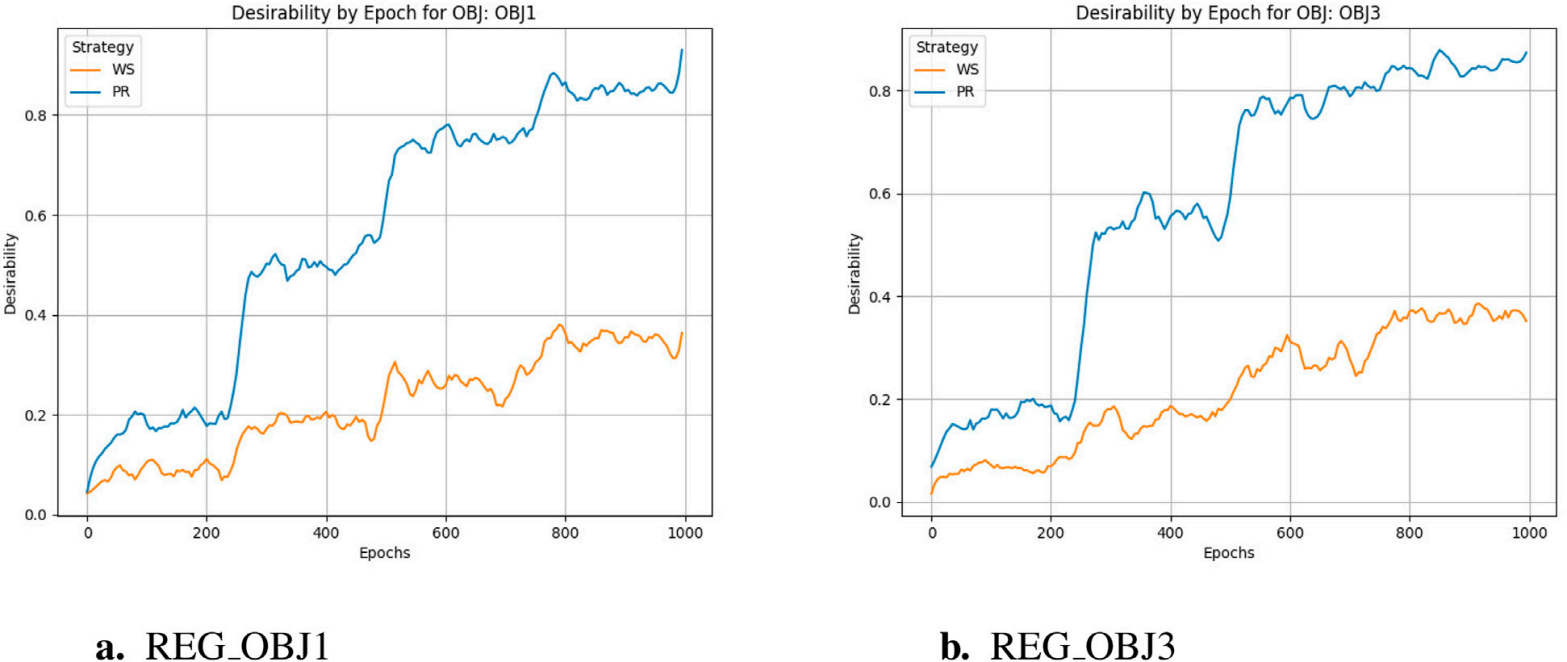
Desirability comparison. **(a)** REG_OBJ1. **(b)** REG_OBJ3.

The training cycle is 1000 for multiple targets and 1500 for specific targets. When applying crossover and variation, crover’s parameters are updated every 250 cycles. REG OBJ1 and REG OBJ3 denote reinforcement-learning objectives targeting A1/A2A and A2A-only selectivity, respectively. As can be seen from Figure 3a, the effectiveness of the Pareto optimization strategy during the training process is notably higher than that of the weighted strategy. Its average performance begins at a higher level and rises more swiftly, ultimately nearing 1.0. In comparison, the weighted strategy’s average performance improves at a slower pace and plateaus around 0.4, which is considerably less than the Pareto optimization strategy’s performance. Consequently, it is evident that the Pareto optimization strategy is superior to the weighted strategy. There were two large jumps in desirability during the initial training, presumably because crover’s parameters were updated to improve the crossover effect in the evolutionary algorithm. From Figure 3b, we can observe the following:the Pareto optimization strategy significantly outperforms the weighted strategy during the training process. Its desirability not only starts at a higher level but also increases more rapidly, eventually exceeding 0.8. In contrast, the desirability of the weighted strategy increases more slowly and stabilizes around 0.4, which is significantly lower than that of the Pareto optimization strategy. This figure clearly demonstrates the performance difference between the two strategies during the training process, with the Pareto optimization strategy showing markedly better performance for the targe. According to speculation, when the Epoch is equal to 750, there should be a significant jump in desirability, but it does not appear in the actual situation, indicating that the improvement of model training has basically achieved enough excellent results, and the update of crover parameters involved in the cross has not significantly improved. However, if we only look at the weight-based scheme, in the optimization of multiple objectives and specific objectives, there is also a small jump in the corresponding nodes, but the improvement rate is much lower than that of the Pareto scheme, indicating that the improvement effect of the evolutionary algorithm is not so obvious in the weighted scheme.

**FIGURE 4:**
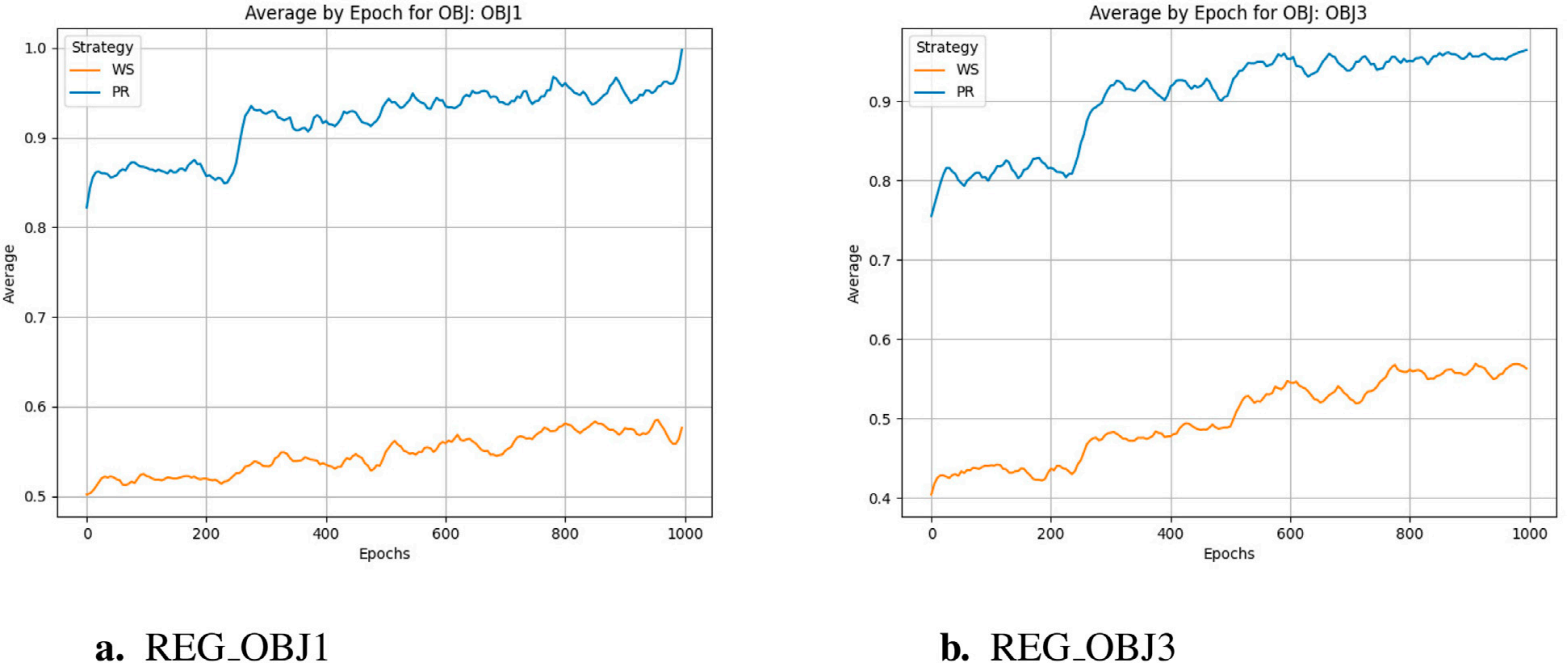
Score comparison. **(a)** REG_OBJ1. **(b)** REG_OBJ3.

As can be seen from Figure 4, in the training process of reinforcement learning, reward scores gradually increase with the increase of training cycle, which is basically consistent with the change trend of acceptability. This shows that in reinforcement learning, no matter based on Pareto frontier or weighted scheme, multi-objective optimization strategy is effective. However, it is worth noting that through the comparison within the figures and observing between Figures 4a,b, it can be found that compared to the latter, the former not only achieved a higher reward score at the end of training but also showed better improvement compared to the beginning of training, and reaches the critical value in fewer training cycles. However, from the trends in the following two graphs, it is reasonable to infer that if the weight-based scheme continues to train, it can also get a relatively reasonable result, but the training period is much longer than the normal requirements.

The final training results are shown in Table 1. In order to reduce errors, we selected the training results of the last 10 training cycles, calculated their average values for comparison, and finally obtained the following results.

**TABLE 1:** Performance comparison.

Optimizing strategy	Objective	Reward score	Effectiveness	Desirability
PR	Multiple target	0.9596	0.9644	0.8665
PR	Specific objective	0.9747	0.9815	0.8903
WS	Multiple target	0.5622	0.9670	0.3833
WS	Specific objective	0.56892	0.9609	0.35652

• WS = Weighted-Sum strategy, a linear aggregation of multi-objective rewards.

• PR = Pareto-Rank strategy, a non-dominated sorting approach that honors the Pareto frontier.

It can be clearly seen that the reward scores obtained by the optimization strategy using Pareto frontier and the feasibility of generating molecules are far superior to the optimization strategy based on weight, which proves that our choice is correct. However, it is worth noting that although the difference is not large, the effectiveness of the former is slightly less than that of the latter. But taking into account other metrics and performance improvements, a small lag in this single area is acceptable.

**FIGURE 5:**
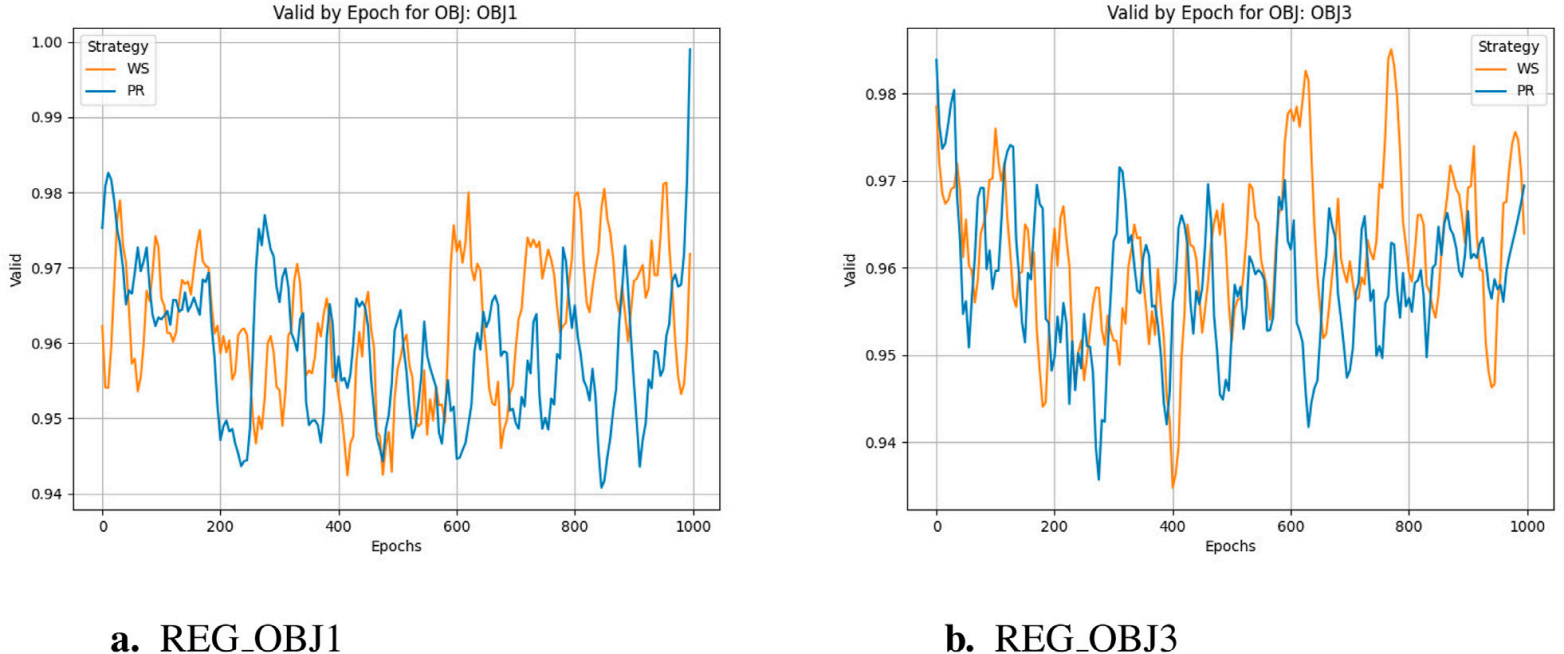
Effectiveness comparison. **(a)** REG_OBJ1. **(b)** REG_OBJ3.

According to the analysis in Figure 5, it can be found that there is little improvement in the effectiveness of the generative model to generate molecules in the reinforcement learning stage. Although in the early stages of training, reinforcement learning can help the model find polymer generation strategies more quickly, as the training progresses, the generative model has been able to generate higher quality molecules, so the effect of reinforcement learning on it is no longer obvious.

### 3.2 Comparative experiment

Above shows that the early pre-training stage is very important in the training process of molecular generative model, and through pre-training, the generative model can generate high-quality molecules faster and more accurately. The role of reinforcement learning in the training of molecular generative models requires more specific analysis and evaluation to determine its impact on the generation of high-quality molecules. After the pre-training is completed, the molecular effectiveness generated by the generative model itself is already close to quite high, and there is not much room for improvement. Although the effectiveness of the model fluctuates somewhat during the training process, most of them are above 0.95, indicating that the pre-training has made the model achieve a very good effect in the generation of molecules, and it is acceptable that the effectiveness is not significantly improved.

**FIGURE 6:**
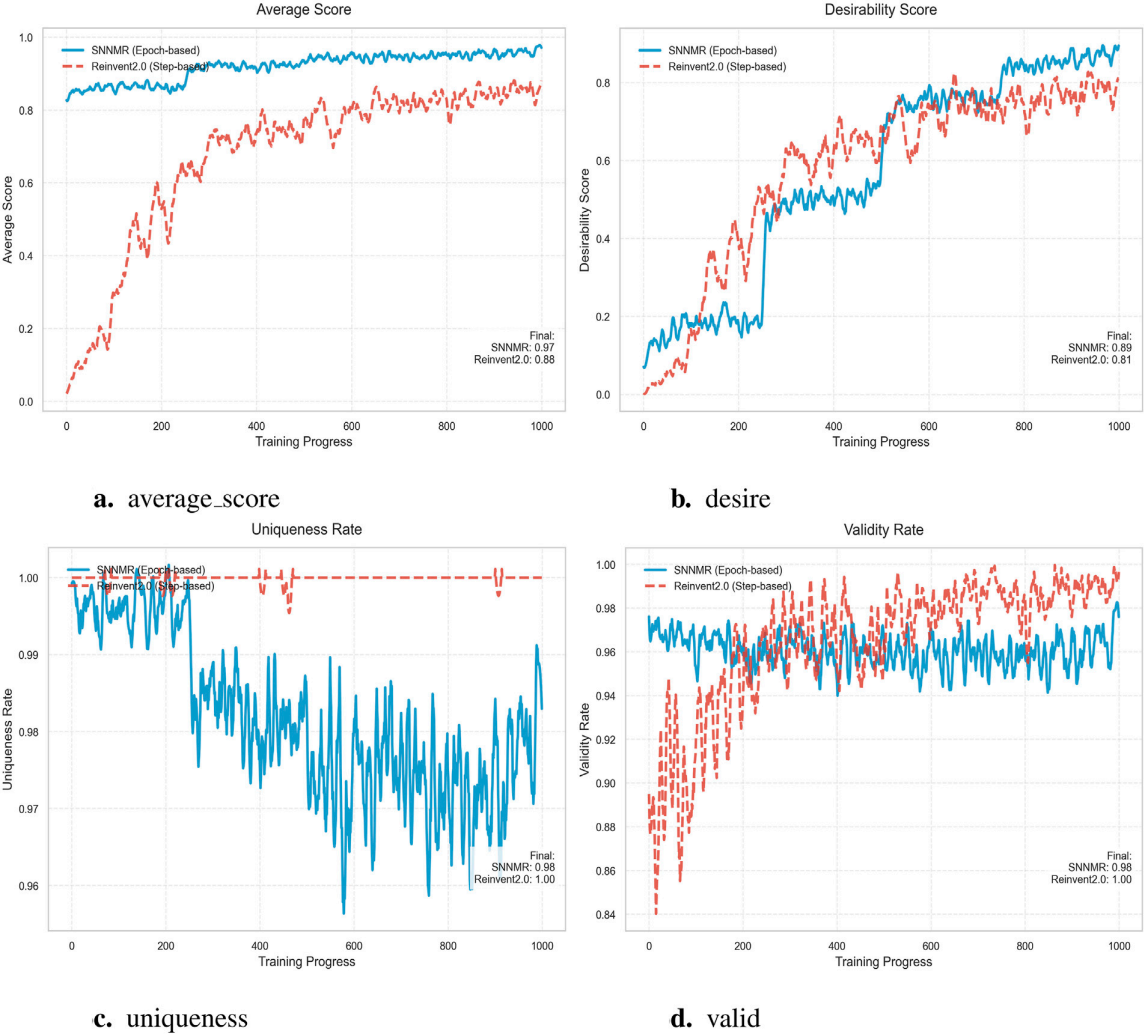
Model Performance comparison. **(a)** average_score. **(b)** desire. **(c)** uniqueness. **(d)** valid.

After in-depth analysis of the data presented in the four subgraphs, we can clearly see the superior advantages of the SNNMR model across a number of key performance indicators. First of all, from the core indicator of average score, SNNMR model performs well in multiple training stages. In Figure 6a, SNNMR has a slightly higher average score (0.97) than Reinvent2.0 (0.88), which initially shows the potential advantages of SNNMR for specific tasks. Looking further at Figure 6b, although the mean score of SNNMR (0.98) is very close to that of Reinvent2.0 (1.00), SNNMR scores change more smoothly over the course of training, showing greater stability and reliability. In Figure 6c, SNNMR’s average score (0.89) is once again higher than that of Reinvent2.0 (0.81), further cementing SNNMR’s lead in performance. Finally, in Figure 6d, the smoothness of the SNNMR score curve once again highlights its stable training process and good generalization, although the average scores of the two are close again. In addition, the stability of SNNMR models during training also deserves special mention. In the four subgraphs, SNNMR score curves show a smooth and stable trend, which indicates that the model can adapt to data changes well in the training process and avoid overfitting or underfitting problems. This stability not only helps to improve the generalization ability of the model, but also reduces the cost of model adjustment and optimization in practical applications. Subsequently, a comparison of the various indicators between SNNMR and GENTRL (Zhavoronkov et al., 2019), Reinvent2.0 (Blaschke et al., 2020), as well as Diff - AMP (Wang et al., 2024) will be conducted respectively.

Comparative analysis demonstrates SNNMR’s superior performance with 98.15 % validity, significantly outperforming GENTRL’s 4.1 % . This high validity ensures most generated molecules meet chemical requirements for direct experimental validation. While SNNMR shows slightly lower novelty (89.5 % vs. 97.56 % ) and uniqueness (98 % vs. 100 % ), this trade-off achieves a more balanced profile with comparable average scores (2.9480 vs. 2.9730). The model’s 2 % duplicate retention enables efficient reuse of known activity data while maintaining sufficient molecular diversity, making it particularly valuable for industrial drug discovery where experimental feasibility and cost efficiency are prioritized.

Comprehensive evaluation of Table 2 shows that SNNMR outperforms Reinvent2.0 across all key metrics. First, its Desire (target-achievement rate) reaches 89.00 % , a 5.44-percentage-point lead over Reinvent2.0’s 84.38 % , indicating significantly better fulfillment of the predefined objectives. Second, the Average Score surges from 0.9041 to 2.9480—more than a three-fold increase—demonstrating a substantial leap in overall optimization quality. Third, VALID (validation accuracy) remains at 98.15 % , within 2 % of Reinvent2.0’s 100 % , confirming that the performance gains do not compromise generalization reliability. Finally, Uniqueness stays high at 98.00 % , only 2 % below Reinvent2.0, preserving sample diversity and deduplication capability. Consequently, SNNMR delivers a quadruple advantage—higher target-achievement rate, higher average performance, high validation accuracy, and high uniqueness—achieving a clear and comprehensive superiority over Reinvent2.0.

**TABLE 2:** Performance comparison.

Model	Average	VALID	Uniqueness
GENTRL	2.9730	0.0410	1.0000
Reinvent2.0	0.9041	1.0000	1.0000
Diff-AMP	3.4580	0.1516	0.1274
SNNMR	2.9480	0.9815	0.9800

• Validity is defined as the ratio of chemically valid molecules among all generated molecules.

• Novelty is measured as the proportion of valid molecules not present in the training dataset.

• Uniqueness quantifies the number of distinct valid molecules generated.

In the comparative experiment between the SNNMR and Diff - AMP models, distinct performance characteristics are observed. The SNNMR model demonstrates a VALID value of 0.9815, indicating a high degree of validity in its outputs, which signifies that the majority of its generated results are effective and accurate. In contrast, the Diff - AMP model has a VALID value of only 0.1516, reflecting a relatively low level of effectiveness and a higher proportion of invalid or inaccurate outputs. Regarding uniqueness, the SNNMR model achieves a Uniqueness value of 0.9800, showcasing a strong ability to produce diverse and non - repetitive results. On the other hand, the Diff - AMP model’s Uniqueness value is a mere 0.1274, indicating a significant lack of uniqueness in its outputs. Although the Diff - AMP model has a higher average value (3.4580) compared to SNNMR’s 2.9480, this higher average may imply greater performance volatility. Overall, the SNNMR model outperforms the Diff - AMP model in terms of both validity and uniqueness, making it a more reliable and versatile choice for the task at hand.

In the comparative study of the GENTRL, Reinvent2.0, Diff-AMP, and SNNMR models, distinct performance characteristics are evident. The GENTRL model, despite achieving a perfect Uniqueness score of 1.0000, suffers from a very low VALID value of 0.0410, which significantly undermines its overall utility. The Reinvent2.0 model, with both VALID and Uniqueness values at 1.0000, demonstrates high levels of effectiveness and diversity, but its relatively low average value of 0.9041 may imply a certain degree of conservatism in performance. The Diff-AMP model has a high average value of 3.4580, yet its low VALID value of 0.1516 and extremely low Uniqueness value of 0.1274 reveal substantial shortcomings in terms of output validity and diversity. In stark contrast, the SNNMR model excels in multiple aspects. It achieves a high VALID value of 0.9815, ensuring a large proportion of valid outputs, and a commendable Uniqueness value of 0.9800, indicating a strong ability to generate diverse results. Moreover, its average value of 2.9480 reflects a stable and well-balanced performance. Overall, the SNNMR model stands out as the most promising approach among the four, as it effectively combines high validity, good uniqueness, and stable performance, making it a superior choice for the task at hand.

### 3.3 Case analysis

In this experiment, our model will conduct screening and validation of natural compounds targeting the Adenosine A2A receptor (A2A receptor), aiming to generate novel molecules from the Data Set and verify their potential as A2A receptor inhibitors.

**FIGURE 7:**
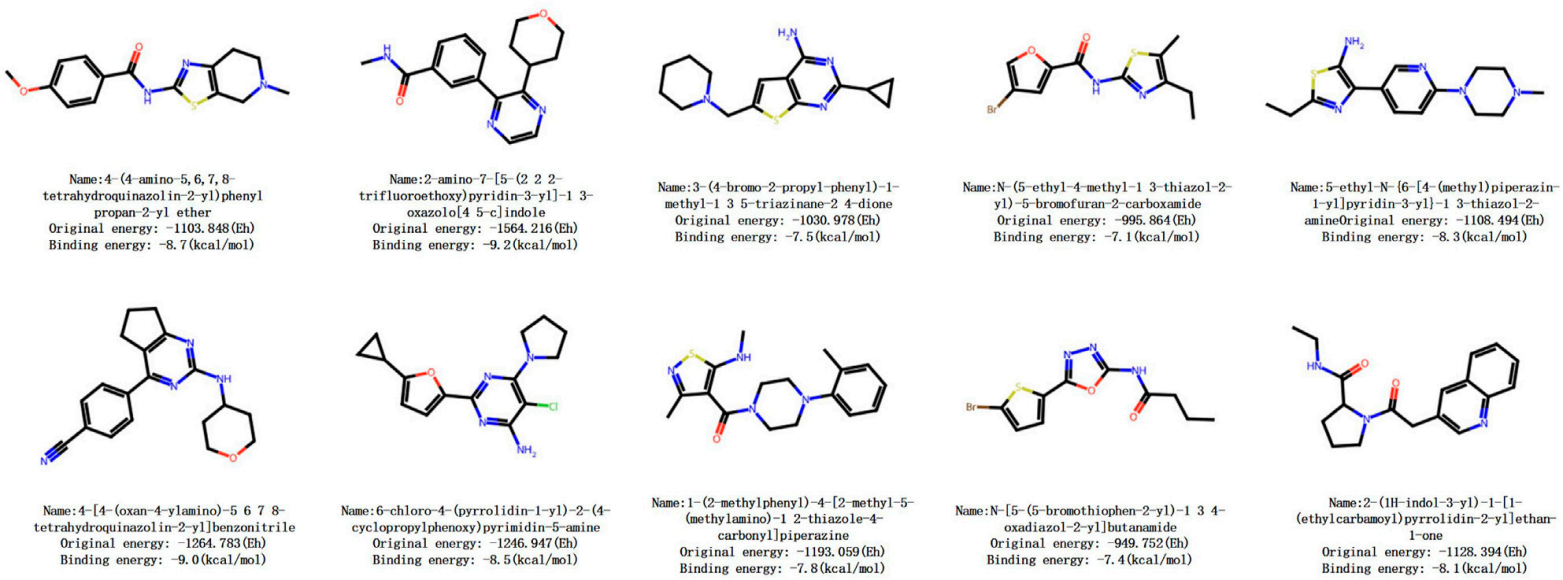
The figure of the top 10 scored molecules.

Firstly, brand-new molecular structures are generated to ensure novelty. Subsequently, based on the reward score, effectiveness, and uniqueness, Pareto optimization ranking is performed using the composite score formula (composite score = (desirability × 0.4) + (reward score × 0.4) + (uniqueness × 0.2)). The top 10 molecules Figure 7 are selected, and a random selection is made for case analysis. Next, the screened molecules undergo 3D structural modeling and are docked with the 3D structure of the A2A receptor to simulate the binding mode. Binding free energy and other indicators are calculated to evaluate the binding situation.

Taking a certain molecule as an example, its binding free energy is −6.18 kcal/mol, indicating spontaneous binding and falling within the acceptable range of drug binding energy (−6 to −12 kcal/mol). It shows a significant improvement of 73 % compared to the other result (−3.56), demonstrating good binding strength. The ligand efficiency of this molecule is −0.29 kcal/mol/heavy atom, which is within the ideal range. The inhibition constant is 29.7 μM , at the upper limit of weak inhibitors, indicating moderate inhibitory ability. The results indicate that this molecule exhibits high activity towards the A2A receptor and can bind to it, suggesting that this compound may have potential therapeutic effects in areas such as tumor immunotherapy.

In conclusion, the preliminary activity of this compound is favorable, indicating that our model is effective and reliable in generating drug molecules and has good prospects for pharmaceutical applications.

## 4 Conclusion

In this study, we present an advanced methodology for drug molecular design based on multi-objective optimization. A robust predictive model was developed using the Random Forest algorithm, which is widely recognized for its efficacy in handling complex datasets. To enhance the generative capabilities, we refined the traditional Recurrent Neural Network (RNN) architecture by integrating Long Short-Term Memory (LSTM) layers and incorporating a self-attention mechanism, thereby significantly improving the model’s ability to capture long-range dependencies and intricate molecular patterns. Additionally, we employed evolutionary algorithms, utilizing crossover, mutation, and selection operations, to iteratively optimize the quality of generated molecular structures. Comprehensive ablation studies were conducted to validate the proposed methodology, with results unequivocally demonstrating the superior performance and effectiveness of our approach in generating high-quality drug-like molecules. Analogous hypervolume-driven multi-objective frameworks have recently been leveraged for antimicrobial peptide discovery, underscoring the broad applicability of our attention-based evolutionary strategy (Wang et al., 2025).

However, we acknowledge that equipment constraints have limited the full potential of our methodologies. Specifically, the stacking of neural networks has prolonged training durations, and the inclusion of multi-head attention modules has necessitated greater memory resources than initially anticipated. Consequently, optimizing the trade-off between training efficiency and performance within constrained computational resources remains a critical research challenge. Additionally, while the evolutionary algorithm inherently exhibits adaptive tendencies towards optimality, its full potential has yet to be fully realized in this context. Our current implementation primarily employs crossover and mutation strategies to enhance molecular diversity. A more comprehensive integration of the evolutionary algorithm with reinforcement learning’s policy gradients holds promise for theoretically superior outcomes. A notable limitation is that the model was trained solely on ChEMBL34, leaving its performance on proprietary or newly released databases unverified; furthermore, all bioactivity and toxicity predictions were generated *in silico*, with no accompanying *in-vitro* or *in-vivo* validation.

Despite these challenges, this project has successfully established a deep learning framework for drug molecular design. The results achieved not only meet the project requirements but also surpass our initial expectations, highlighting the feasibility and potential of our methodology. This underscores the significant promise of our approach in advancing the field of drug discovery and molecular design.

## Data Availability

The original contributions presented in the study are included in the article/supplementary material, further inquiries can be directed to the corresponding authors.
